# Potential Role for *Mycobacterium tuberculosis* Specific IL-2 and IFN-γ Responses in Discriminating between Latent Infection and Active Disease after Long-Term Stimulation

**DOI:** 10.1371/journal.pone.0166501

**Published:** 2016-12-29

**Authors:** Qin Sun, Wei Wei, Wei Sha

**Affiliations:** Clinic and Research Center of Tuberculosis, Shanghai Key Laboratory of Tuberculosis, Shanghai Pulmonary Hospital, Tongji University School of Medicine, Shanghai, China; Suzhou University, CHINA

## Abstract

Interferon gamma release assays (IGRAs) could accurately diagnose *Mycobacterium tuberculosis* (*M*.*tuberculosis*) infection. However, these assays do not discriminate between latent tuberculosis infection (LTBI) and active tuberculosis disease (ATB). Here, a total of 177 subjects, including 65 patients with ATB, 43 subjects with LTBI, and 69 TB-uninfected controls (CON group) were enrolled. The concentration of IFN-γ, IP-10, and IL-2 was determined in peripheral blood mononuclear cells (PBMCs) after short-term (24h) or long-term (72h) stimulation with TB antigens including ESAT-6/CFP-10 (EC) and purified protein derivative (PPD).EC-stimulated IL-2 and gamma interferon-inducible protein 10 (IP-10) release (24h and 72h) showed a good diagnostic performance in distinguishing between TB-infected and TB-uninfected individuals, but failed to discriminate between ATB and LTBI. After 72h of incubation, the release of IL-2 was higher in LTBI patients after stimulation with EC and PPD. The PPD-stimulated IL-2/IFN-γ ratio after 72h incubation had the diagnostic potential to discriminate between ATB and LTBI, with a sensitivity of 90.8% and a specificity of 97.7%. In addition, these new biomarkers, combined with T-SPOT test in a two-step strategy, were validated with high levels of accuracy in a prospective clinical-based cohort. Collectively, the PPD-stimulated IL-2/IFN-γ ratio after long-term incubation may be an alternative diagnostic biomarker in distinguishing between active TB patients and subjects with latent infection.

## 1. Introduction

Tuberculosis (TB) remains a serious global health problem with nearly 10 million new cases and 1.5 million deaths every year (WHO TB report 2015). TB now ranks alongside HIV as a leading cause of death worldwide. Furthermore, it is estimated that two billion people live with latent *M*.*tuberculosis* infection (LTBI), which may represent a potential source of future active tuberculosis[1,2]. However, despite intensive research effort, rapid and accurate diagnosis of active tuberculosis remains difficult[3,4]. New tuberculosis biomarkers are therefore urgently needed[3,4].

The interferon-gamma release assay (IGRA) has been widely used and is recognized as the most important advancement in TB immunodiagnosis[5,6]. It detects the cell-mediated immune response by measuring the *in vitro* IFN-γ production in response to stimulation with *M*.*tuberculosis*-specific antigens[5,6]. There are two commercially available kits: QuantiFERON-TB Gold In-Tube Test (QFT) from Cellestis/Qiagen and T-SPOT.TB test (T-SPOT) from Oxford Immunotec. The *M*.*tuberulosis*-specific antigens used in the IGRAs include the early secreted antigenic target-6 (ESAT-6), the culture filtrate protein-10 (CFP-10), and the TB7.7 (Rv2654) antigen. IGRAs have been systematically reviewed and shown to have higher sensitivity and specificity for *M*.*tuberculosis* infection compared with the tuberculin skin test (TST)[7,8]. However, a positive IGRA result does not distinguish between active TB and latent infection, which limits its use for routine diagnosis of active TB in areas with high TB incidence[9]. Thus, identification of biomarkers that can rapidly differentiate between active disease and latent infection would be a major breakthrough.

Beside IFN-γ, other cytokines released by *M*.*tuberculosis*-specific antigen stimulation were investigated as alternative or adjunct biomarkers of *M*.*tuberculosis* infection. Several studies have demonstrated that the IL-2 response to *M*.*tuberculosis*-specific antigens is significantly higher in active TB patients, suggesting that IL-2 could be a potential infection biomarker[10,11]. Gamma interferon-inducible protein 10 (IP-10) was shown to be an alternative diagnostic biomarker to IFN-γ and is produced in large amounts[12]. However, few studies evaluated the performance of IL-2 and IP-10 in subjects with latent infection or at high risk of TB exposure, especially in a TB-endemic and BCG-vaccinated area. In addition, a retrospective study by Biselli *et al*., indicated that IL-2 release in response to long-term incubation with *M*.*tuberculosis*-specific antigens increased only in subjects with LTBI. This could potentially exclude healthy individuals and discriminate between active TB and LTBI[13]. Sincelong-term incubationmay allow a better stimulation of *M*.*tuberculosis*-specific central memory T-cells and ultimately result in the release of various cytokines[4], the diagnostic performance of other candidate biomarkers stimulated for prolonged incubation time requires further investigation.

The aim of our study was to develop an improved T-cell based immunodiagnostic assay with higher performance in discriminating ATB from LTBI. Therefore, we first conducted a cross-sectional study to investigate the diagnostic value of IFN-γ, IP-10, and IL-2 in PBMCs stimulated with *M*.*tuberculosis*-specific antigens in identifying patients at different stages of disease. The diagnostic performance of cytokines following short-term (24h) or long-term (72h) stimulation was also investigated. Selected biomarkers were further validated for their ability to discriminate between ATB and LTBI in a clinical group.

## 2. Methods

### 2.1. Study groups

Patients in our study were recruited at the Shanghai Pulmonary Hospital between 2013 and 2015. We investigated the diagnostic performance of cytokines in two study groups. General information and clinical characteristics of the subjects are summarized in Table 1. Group I comprised a total of 177 subjects consisting of 65 active TB patients (ATB), 43 subjects with latent tuberculosis infection (LTBI), and 69 TB uninfected controls (CON). ATB patients were diagnosed based on clinical manifestations, laboratory isolation on smear of acid fast bacilli (AFB), positive culture of *M*.*tuberculosis*, and classical radiological images. To minimize the effects of anti-TB treatment on T-cell responses, all the samples were collected prior to the initiation of therapy. Only patients receiving standard anti-TB therapy for <1 weeks were included in the study.

**Table 1:** Demographic and clinical characteristics of the study population.

Characteristic	Study group I			Study group II	
	ATB	LTBI	CON	ATB	NTB
**Total no.**	65	43	69	39	73
**Male, n(%)**	41(63.1)	23(53.5)	39(56.5)	22(56.4)	38(52.1)
**Median age(range),years**	44(19–71)	49(25–67)	41(18–66)	36(21–73)	41(19–77)
**HIV positive, n(%)**	2(3.1)	0(0)	0(0)	1(2.6)	2(2.7)
**Diabetes, n(%)**	10(15.4)	6(14.0)	8(11.6)	4(10.3)	11(15.1)
**BCG status**					
** Unvaccinated**	19(29.2)	9(20.9)	18(26.1)	11(28.2)	17(23.3)
** Vaccinated**	42(64.6)	31(72.1)	46(66.7)	25(64.1)	50(68.5)
** Unknown**	4(6.2)	3(7.0)	5(7.2)	3(7.7)	6(8.2)
**T-SPOT results**					
** Negative**	6(9.2)	0(0)	69(100)	3(7.7)	43(58.9)
** Positive**	59(90.8)	43(100)	0(0)	36(92.3)	30(41.1)

Subjects with LTBI (LTBI group) were recruited from household contacts of ATB patients. T-SPOT tests were performed on all the subjects and LTBI was defined by a positive response to the QuantiFERON-TB Gold In Tube test, without signs of active disease.

The 69 TB uninfected controls (CON group) were recruited from subjects that came for an unrelated health examination to the hospital. T-SPOT tests were also performed on all subjects and only those with negative T-SPOT results, no clinical and radiographic evidence of ATB, and no known history of exposure to TB were enrolled.

Group II consisted of 112 subjects suspected of active TB in a prospective cohort. All subjects presented with clinical or radiographic characteristics consistent with active TB. They were finally diagnosed as active TB if a positive *M*.*tuberculosis* culture and/or positive smear of acid AFB was obtained (ATB group). Those who did not meet the criteria of active TB were diagnosed as subjects without active TB disease (NTB group).

Experiments and the protocols in this study were approved by the Ethics Review Board (ERB) of Shanghai Pulmonary Hospital and Tongji University School of Medicine (Shanghai, China).The written-informed consent was obtained from each enrolled individual. All investigations were conducted according to the principles expressed in the Declaration of Helsinki as well as national/international regulations.

### 2.2. Isolation of PBMCs and T-SPOT test

Peripheral blood (10 mL) was drawn from the median cubital vein of the antecubital fossa of each participant and collected in heparinized vacutainer tubes (Becton Dickinson, USA). Peripheral blood mononuclear cells (PBMCs) were isolated from heparinized venous blood by Ficoll-Paque centrifugation within 6 hours of collection. Trypan blue non-stained cells were counted using a Countess Automated Cell Counter (Invitrogen, USA) and the number was adjusted to a density of 2.5×10^6^ cells/mL. T-SPOT.TB kit (Oxford Immunotec Ltd., Oxford, UK) was employed to identify *M*.*tuberculosis* infection, including latent and active *M*.*tuberculosis* infection, and was utilized as per manufacturer’s instructions. The test result of T-SPOT.TB assay was considered positive if either or both Panel A (containing peptide antigens derived from ESAT-6) and Panel B (containing peptide antigens derived from CFP-10) had six or more spots than the negative control, and the number was at least twice that of the negative control. Spots were read using the ELISPOT plate reader (AID-Gmb-H, Germany).

### 2.3. *M*.*tuberculosis*-specific antigen stimulated cytokine release assays

PBMCs were cultured at a density of 1.25x10^6^ cells in 800 μl of AIM-V media (Invitrogen Life Technologies, USA) and incubated with ESAT-6 and CFP-10 (each at a concentration of 10 μg/mL; Sangon Biotech, Shanghai, China), purified protein derivative (PPD, 20 μg/mL; RT50; Statens Serum Institute, Copenhagen, Denmark), or without stimulant (negative control). Following incubation at 37°C for 24h (short time) and 72h (long time), supernatants were harvested and stored at -80°C for batch analysis.

### 2.4. IFN-γ, IP-10 and IL-2 measurement/Cytokine analysis

The cytokine concentrations were measured in supernatants from PBMCs stimulated with *M*.*tuberuculosis*-specific antigens. The Biolegend ELISA kit was used to determine IFN-γ, IP-10, and IL-2 levels according to the manufacturer’s instructions. Samples were diluted 1:2 for IFN-γ and IL-2, and 1:10 for IP-10 determination. All samples were tested in duplicates and results were expressed in pg/mL.

### 2.5. Statistical analysis

*M*.*tuberculosis*-specific antigen stimulated cytokine response was defined as the cytokine concentration in the supernatant of unstimulated PBMCs subtracted from the cytokine concentration in the supernatant of ESAT-6/CFP-10 or PPD stimulated PBMCs, as detected by ELISA. GraphPad Prism 6 (GraphPad Software, San Diego, CA) was applied for statistical analysis and graphic depiction of data. Comparison of the biomarker distribution between the two groups was performed using the Mann-Whitney U test. Differences among three groups were compared using Kruskal-Wallis tests, followed by a Dunn’s post-test to correct for multiple comparisons. A *P*-value of <0.05 was considered statisticallysignificant. Receiver operating characteristics (ROC) curve analysis was performed to evaluate the diagnostic efficacy of the biomarkers. The cut-off values were estimated at various sensitivities and specificities and determined at the maximum Youden’s index (YI), *i*.*e*. sensitivity+ specificity -1[14].

## 3. Results

### 3.1. Characteristics of enrolled individuals

A total of 177 subjects were recruited in study group I for biomarker screening. Characteristics of enrolled subjects were summarized in Table 1. There were no significant differences among the three groups regarding age or gender.The BCG vaccination rate was 64.6% in ATB, 72.1% in LTBI, and 66.7% in control (“CON”) group, with no significant difference among them.

The 65 active TB patients comprised 58 with pulmonary TB, 2 with spinal TB, 2 with lymph node TB, and 3 with TB meningitis. For the active TB patients, 25 patients had a positive sputum culture for *M*. *tuberculosis*, 7 patients had a positive acid-fast bacillus (AFB) smear, 28 patients were positive for both markers, and 5 patients were negative for both markers but were diagnosed with TB based on positive histopathological findings, clinical manifestations, and chest radiography. The positive rate of T-SPOT was 90.8% (59/65) in the ATB group.

### 3.2. *M*.*tuberculosis*-specific cytokine responses of IFN-γ, IP-10, and IL-2 after short-termstimulation

**Fig 1:**
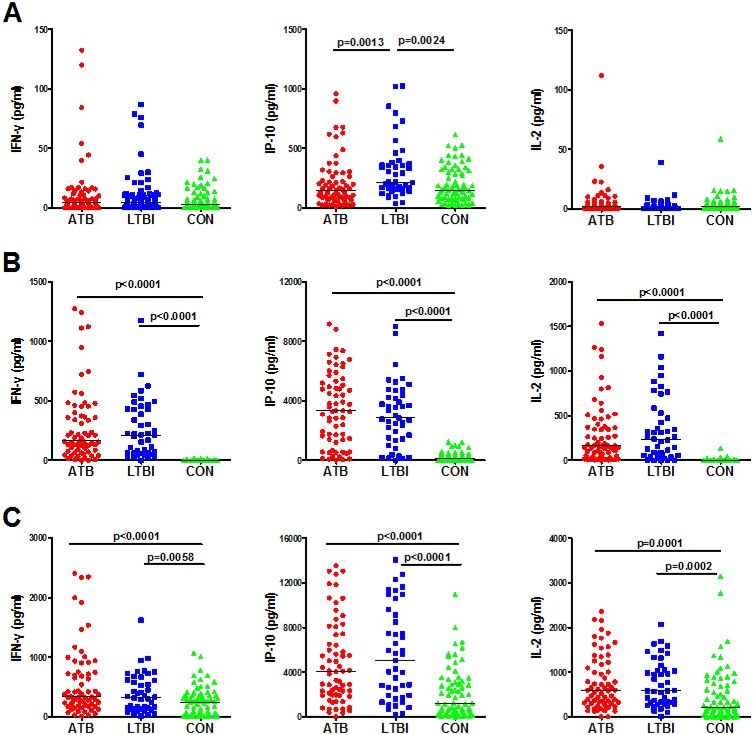
Levels of IFN-γ, IP-10, and IL-2 in active TB patients (ATB), subjects with latent TB infection (LTBI), and TB-uninfected controls (CON) groups, after short-term (24h) incubation with non-stimulated control (A), ESAT-6/CFP-10 (B), and PPD (C). Each data point represents the concentration observed in the non-stimulated level (A) or following antigen stimulation minus the concentration observed in non-stimulated controls (B and C).

In order to evaluate the diagnostic potential of the cytokines, the PBMCs from ATB, LTBI, and CON groups were first stimulated with EC or PPD for 24h. Both antigen-stimulated and unstimulated cytokine responses were analyzed in the study. Levels of IFN-γ and IL-2 were relatively low in unstimulated samples from all three groups and no significant differences were observed (Fig 1A). However, the unstimulated IP-10 release was significantly higher in LTBI group than in both the ATB and CON groups (p = 0.0013 and p = 0.0024 respectively; Fig 1A).After EC stimulation, the release of IFN-γ, IP-10, and IL-2 was significantly higher in the ATB and LTBI groups than that in CON group (Fig 1B). The PPD stimulated IFN-γ, IP-10, and IL-2 responses were also significantly higher in ATB and LTBI group than CON group (Fig 1C). However in CON group, there were relatively higher responses for the three cytokines after PPD stimulation than those after EC stimulation and all the differences were significant (p<0.0001).

Next, we determined whether any cytokines were differentially expressed between ATB and LTBI groups. As expected, IFN-γ release was not significantly different between the two groups, either stimulated with EC or PPD. For IL-2 and IP-10 responses in the short-term assay, there was also no significant difference observed between ATB and LTBI after ECor PPD stimulation.

### 3.3. *M*.*tuberculosis*-specific cytokine responses of IFN-γ, IP-10, and IL-2 after long-term stimulation

**Fig 2:**
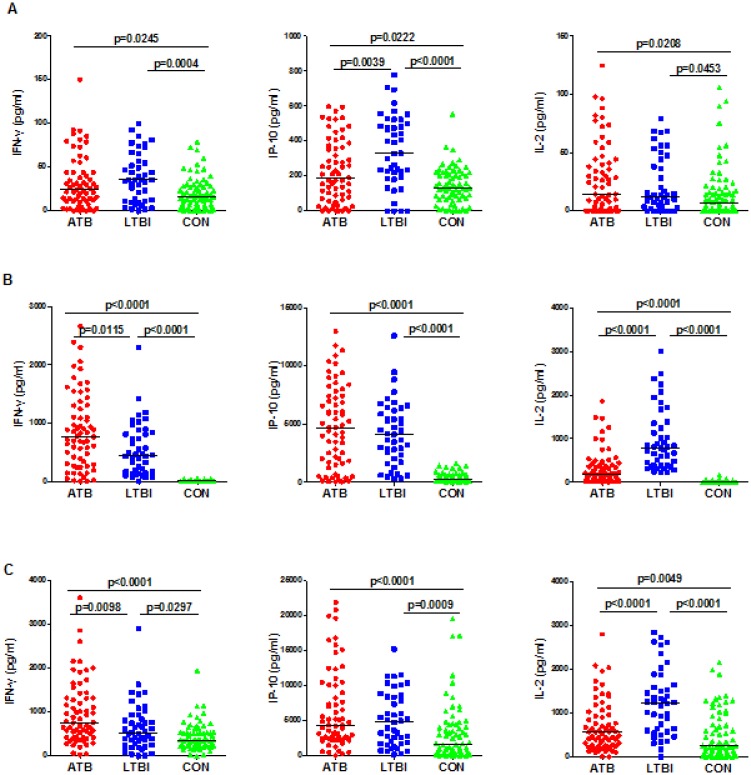
Levels of IFN-γ, IP-10 and IL-2 in active TB patients (ATB), subjects with latent TB infection (LTBI), and TB-uninfected controls (CON) groups, after long-term (72h) incubation with unstimulated control (A), ESAT-6/CFP-10 (B), and PPD (C). Each data point represents the concentration observed in the unstimulated level (A) or following antigen stimulation minus the concentration observed in unstimulated controls (B and C).

We also analyzed the cytokine responses after stimulation with EC or PPD for a longer time of incubation (72h). Similarly, the IFN-γ, IP-10, and IL-2 release was significantly higher in the ATB and LTBI groups than that in the CON group after stimulation with either ECor PPD (Fig 2B and 2C). Then we analyzed the cytokine response between the ATB and LTBI groups and determined that LTBI subjects secreted significantly higher levels of IL-2 than subjects in the ATB groupwith EC or PPD stimulation (p<0.0001). In contrast, the IFN-γ response was significantly higher in the ATB group stimulated with EC (p = 0.0115) or PPD (p = 0.0098). IP-10 response showed no significant difference between the LTBI and ATB groups.

Unstimulated PMBCs from CON subjects secreted significantly less IFN-γ and IL-2 than those from ATB and LTBI subjects (Fig 2A). Interestingly, we observed that the unstimulated release of IP-10 was as follows, from high to low: LTBI, ATB and CON, and differences between each two groups were significant.

### 3.4. Diagnostic performance of the cytokine response to discriminate between TB-infected and CON group

To determine potential diagnostic biomarkers, ROC analyses were performed to evaluate the diagnostic performance of cytokines responses found to have discriminatory ability in the previous analyses. For this purpose, we first pooled data collected from subjects in the ATB and LTBI groups into a single TB-infected group (TBI group) and compared them with data from the CON group. The results of the ROC analyses between TBI and CON groups were shown in Table 2. For both short-term and long-term assay, IFN-γ response stimulated by EC achieved the highest AUC of 0.9752 and 0.9697, respectively. EC stimulated IL-2 and IP-10 showed high AUCafter 24h or 72h of incubation, which were close to those of IFN-γ with no significant difference in AUC between each pair (Table 2). The results strongly support the diagnostic potential of IP-10 and IL-2 to discriminate between TB-infected and TB-uninfected individuals. Notably, after PPD stimulation, the AUCs of the three cytokines were all significantly lower than those stimulated by EC (p<0.0001).

**Table 2:** ROC analysis for cytokines in discriminating TB infected vs. uninfected group.

Stimulant	Cytokine	TBI (ATB+LTBI)^a^	CON^a^	AUC(95%CI)	P-value	Cut-off (pg/ml)^b^	Sensitivity%	Specificity%
**24h**								
**Unstimulated**	**IFN-γ**	0.7(0–3.1)	1.5(0–4.6)	0.5325(0.4449–0.6201)	0.4667	—	—	—
	**IP-10**	180.7(97.3–340.9)	150.4(82.4–318.5)	0.5541(0.4679–0.6403)	0.2255	—	—	—
	**IL-2**	0.7(0–3.7)	1.4(0–5.6)	0.5298(0.4423–0.6172)	0.5043	—	—	—
**ESAT-6/CFP-10**	**IFN-γ**	195.1(72.3–428.6)	0.3(0–3.1)^c^	0.9752(0.9507–0.9997)	<0.0001	18.5	94.4	100
	**IP-10**	3257.3(1409.2–4824.3)	98.7(17.4–317.5)^c^	0.9282(0.8911–0.9685)	<0.0001	1279.5	77.8	100
	**IL-2**	169.0(51.6–451.6)	2.1(0.0–7.8)^c^	0.9298(0.8911–0.9685)	<0.0001	20.2	84.3	94.2
**PPD**	**IFN-γ**	334.0(169.5–710.4)	242.7(15.7–367.4)^c^	0.6797(0.5995–0.7599)^e^	<0.0001	88.9	88.9	39.1
	**IP-10**	4106.2(1916.0–7898.7)	1220.3(345.1–3123.2)^c^	0.7560(0.6847–0.8273)^e^	<0.0001	1257.3	86.1	53.6
	**IL-2**	584.6(303.9–1139.0)	215.7(67.4–727.3)^c^	0.6987(0.6163–0.7810)^e^	<0.0001	227.5	84.3	53.62
**72h**								
**Unstimulated**	**IFN-γ**	29.7(11.2–51.1)	16.5(5.1–28.3)^d^	0.6467(0.5674–0.7261)	0.0014	30.7	48.2	79.7
	**IP-10**	238.6(106.6–447.5)	129.6(68.9–216.3)^c^	0.6809(0.6034–0.7584)	<0.0001	249.1	48.2	89.9
	**IL-2**	17.4(2.3–54.3)	9.4(0.0–25.6)^d^	0.6027(0.5185–0.6807)	0.0201	34.6	37.0	84.1
**ESAT-6 /CFP-10**	**IFN-γ**	648.7(243.1–1038.2)	11.2(1.9–18.8)^c^	0.9697(0.9410–0.9983)	<0.0001	58.4	94.4	100
	**IP-10**	4353.5(1772.3–6703.7)	231.7(154.7–542.2)^c^	0.9147(0.8717–0.9576)	<0.0001	1472.2	79.6	98.6
	**IL-2**	275.9(109.7–625.4)	7.5(2.9–14.9)^c^	0.9616(0.9365–0.9867)	<0.0001	46.3	87.0	95.7
**PPD**	**IFN-γ**	747.6(366.8–1143.2)	356.8(228.1–537.5)^c^	0.7052(0.6293–0.7811)^e^	<0.0001	551.8	59.3	78.3
	**IP-10**	4561.5(2288.3–8828.2)	157.1(517.0–4454.2)^c^	0.7050(0.6252–0.7848)^e^	<0.0001	1911.3	81.5	55.1
	**IL-2**	681.9(364.3–1359.2)	255.7(73.3–806.8)^c^	0.7078(0.6283–0.7873)^e^	<0.0001	355.2	76.9	59.4

^a^ Data are presented as median concentration in pg/ml (interquartile range).

^b^ The cut-off values are determined at the maximum Youden’s index (YI), *i*.*e*. sensitivity+ specificity -1.

Statistical significance was determined versus TBI group,

^c^ p<0.0001;

^d^ 0.0001≤p<0.001. (Mann Whitney test)

Statistical significance was determined versus AUC stimulated by ESAT-6/CFP-10,

^e^ p<0.0001.

AUC = Area under the ROC curve.

### 3.5. Diagnostic performance of the cytokine response to discriminate between ATB and LTBI group

Given that current IGRAs assays perform well at identifying the TB-infected individuals (including active and latent TB), we next removed the CON group from consideration and focused on identifying biomarkers that could discriminate between ATB and LTBI. The cytokines differentially expressed between ATB and LTBI groups were selected and ROC analyses were performed to evaluate their diagnostic potential. The results wereshown in Table 3. After long-term incubation, PPD stimulated IL-2 achieved the highest AUC (0.8547; 95%CI: 0.7735–0.9159) and correctly classified 63.1% and 97.7% of the participants in ATB and LTBI group, respectively. In EC-stimulated samples, IL-2 response also correctly classified 70.8% and 76.7% of the participants, respectively. PPD-stimulated IFN-γ response also achieved an AUC of 0.6455 (95%CI: 0.5419–0.7491) with a sensitivity of 44.6% and specificity of 79.1% (Table 3).

**Table 3:** ROC analysis for cytokines in discriminating ATB from LTBI.

Stimulant	Cytokine	P-value	AUC (95%CI)	Cut-off (pg/ml)	Sensitivity %	Specificity %
**24h**						
**Unstimulated**	IP-10	0.0013	0.6837(0.5843–0.7832)	118.8	44.6	88.4
**72h**						
**Unstimulated**	IP-10	0.0048	0.6610(0.5548–0.7672)	176.9	49.2	81.4
**ESAT-6/CFP-10**	IFN-γ	0.0115	0.6438(0.5402–0.7473)	574.6	65.2	62.8
**ESAT-6/CFP-10**	IL-2	<0.0001	0.8259(0.6588–0.8731)	266.3	70.8	76.7
**PPD**	IFN-γ	0.0097	0.6455(0.5419–0.7491)	936.3	44.6	79.1
**PPD**	IL-2	<0.0001	0.8547(0.7735–0.9159)	329.1	63.1	97.7

**Fig 3:**
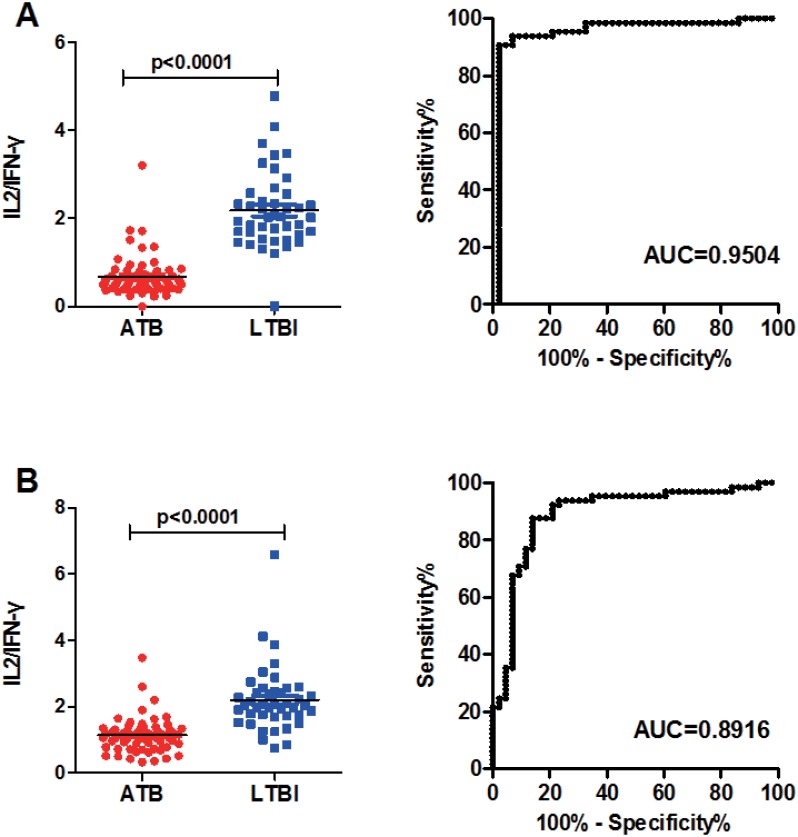
IL-2/IFN-γ ratio in subjects with active TB (ATB group) and in subjects with latent TB infection (LTBI group) after long-term stimulation by PPD (A) and ESAT-6/CFP-10 (B). Horizontal lines indicate the median IL-2/IFN-γ ratio. The ROC analysis use subjects with active TB as patients and subjects with latent TB as controls. The ROC curve and AUC are also shown.

Since the distribution of PPD-stimulated IL-2 and IFN-γ response after 72h of incubation was significantly different between ATB and LTBI groups, but with opposite trends (IL-2 response was higher in LTBI group and IFN-γ response was higher in ATB group), the IL-2/IFN-γ ratios in ATB and LTBI groups were calculated to see if they could improve the diagnostic performance compared with either alone.The results showed that the PPD-stimulated IL-2/IFN-γ ratio was significantly higher in LTBI than ATB group (Fig 3A). The AUC was 0.9504 (95%CI: 0.8979–1.003,p<0.0001) by ROC analysis, and the threshold of 1.14 gave a sensitivity of 90.8% and a specificity of 97.7% for ATB. EC-stimulated IL-2/IFN-γ ratio also showed an AUC of 0.8916 (95%CI: 0.8231–0.9601, p<0.0001; Fig 3B) with a sensitivity of 87.7% and specificity of 86.1% (cut-off value: 1.47). The AUC of EC-stimulated IL-2/IFN-γ ratio was significantly lower than that stimulated by PPD (p<0.001), thus we decided to select PPD-stimulated IL-2/IFN-γ ratio as candidate biomarker for further verification.

### 3.6. Clinical validation of biomarkers in a cohort of active TB suspects

**Fig 4:**
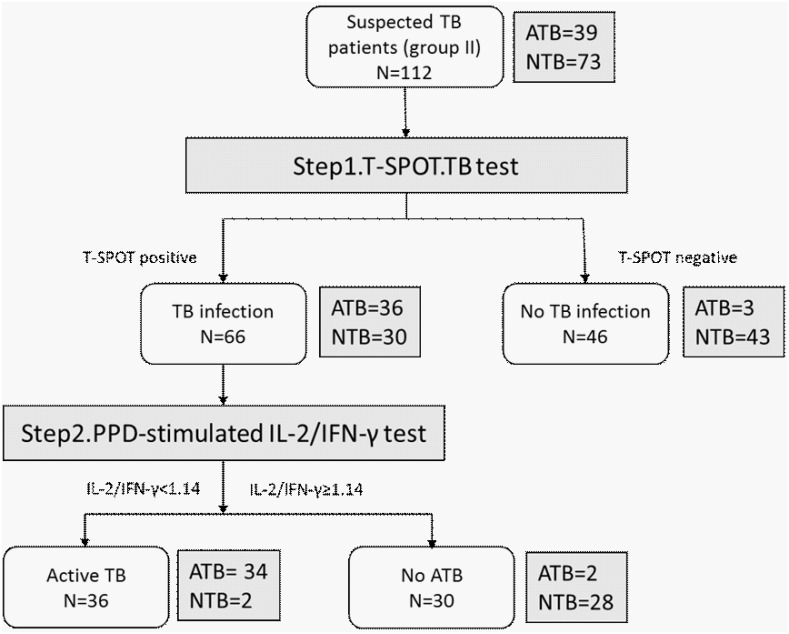
Diagnostic results and strategy for detecting active TB in the validation group. TB suspects were first tested with T-SPOT in step 1, then T-SPOT positive subjects were tested with long-term (72h) PPD-stimulated IFN-γ/IL-2 assays as step 2. The positive results of the two-step assay were defined when both test was positive and a negative result when either negative. The gold standard for ATB was based on positive *M*.*tuberculosis* culture or/and positive acid-fast bacillus smear (ATB group).The subjects without active TB were defined as NTB group.

In order to validate the diagnostic performance of the PPD-stimulated IL-2/IFN-γ ratio, we recruited 112 patients suspected of TB infection, in a clinical-oriented study group (group II). A two-step diagnostic strategy was used: first, T-SPOT tests to detect *M*.*tuberculosis* infection were performed for all the subjects; Second, T-SPOT positive subjects were tested for PPD-stimulated IFN-γ/IL-2 ratio to differentiate between ATB and LTBI. The diagnostic procedure and results were shown in Fig 4. According to the final diagnosis of the 112 subjects in group II, 39 were diagnosed as active TB (12 patients only had a positive culture for *M*.*tuberculosis*, 6 patients only had a positive AFB smear, and 21 patients were positive for both). The sensitivity and specificity were calculated when active TB patients (ATB group) were defined as patients, and subjects without active TB (NTB group) were defined as controls. The T-SPOT test alone achieved a sensitivity of 92.3% and a specificity of 58.9%. When combined with the PPD stimulated IL-2/IFN-γ ratio using the cut-off established before, it achieved a sensitivity of 87.1% and a specificity of 97.3%. The specificity of the combined test was significantly higher than that of the T-SPOT.*TB* test alone (p<0.0001).

## 4. Discussions

Antigen-specific memory T-cells can be subdivided into effector memory T-cells (TEM) and central memory T-cells (TCM)[15]. TEM cells express receptors that enable them to migrate to the inflamed peripheral tissues and differentiate directly into effector cells. These cells can then be detected by measuring the IFN-γ release in short-term incubation assays, using whole blood or PBMCs stimulated with *M*. *tuberculosis* antigens. In contrast, TCM cells are generally thought to be long-lived and can serve as the precursors for effector T-cells in recall responses, which would require longer-term stimulation assays[4]. Therefore, the different incubation periods may result in investigation of different memory T-cells and cytokine profiles. Compared with short-time stimulation assays, more TCM cells could be activated if the incubation time were prolonged. These two subsets of circulating memory T-cells might be participating in different types of immune responses and therefore, have different effector capacities and produce different cytokine profiles[15]. It has been proven that upon activation, TCM cells produce only IL-2, while the TEM subsets produce high levels of IFN-γ but moderately reduced levels of IL-2[15]. This can explain why in our study the cytokine response pattern of IL-2 after 72h stimulation was distinct from those after 24h, and showed a potential diagnostic value for discriminating between ATB and LTBI.

IP-10 has been proven by several studies to be an alternative biomarker for TB infection and IP-10 based tests appear to perform comparably to the current IGRAs[12]. In young children and HIV infected patients with low CD4 count, IP-10 was shown to reduce the incidence of indeterminate results, given its high levels[16]. Our results were consistent with these studies and showed that ESAT-6/CFP-10-stimulated IP-10 had highest AUC in detecting TB infection, but failed to discriminate between ATB and LTBI[11,17,18]. However, unlike IFN-γ and IL-2, the baseline IP-10 level was found to be significantly higher in the LTBI group than in the ATB and CON groups. Similar trends were also observed in several other studies[11,19,20]. This may indicate a protective role of IP-10 in the ongoing control of subclinical infection with *M*.*tuberculosis* in LTBI patients. In our study, the baseline IP-10 showed an AUC of 0.6837 (95% CI) in discriminating between ATB and LTBI, which could not be used alone as an alternative marker. Further studies evaluating the diagnostic performance of IP-10 combined with other biomarkers are still needed.

We show that PPD-stimulated IL-2 and IFN-γ have potential value for discriminating between ATB and LTBI. However, neither of the two cytokines could be used alone as an alternative diagnostic marker. In contrast, the use of cytokine ratios had a stronger discriminatory power in diagnosing different stages of TB infection. IFN-γ/IL-2 ratios in serum showed a potential diagnostic value for detecting extra-pulmonary tuberculosis[21]. Another prospective clinical study also demonstrated that a relative shift from IL-2 towards IFN-γ production in T-cells was associated with active TB[22]. Our results also indicated that PPD-stimulated IL-2/IFN-γ ratio after 72h stimulation was the most accurate discriminator between ATB and LTBI, among all the biomarkers. Differences in IL-2/IFN-γ ratio observed in our study are in agreement with previous studies, which indicated that the frequency of IL-2-secreting and IL-2/IFN-γ-dual-secreting CD^4+^ central memory T-cells increased and the number of IFN-γ-secreting effector memory T-cells was reduced in non-active TB compared with active TB patients[23,24,25]. These changes in cytokine profiles could be associated with the bacterial loads specific for different infection stages. In non-active or successfully treated TB patients, an elevated IL-2 response may be a consequence of the expansion of central memory T-cells, caused by a reduction of *M*.*tuberculosis* antigen loads[26].

In our study, the difference in IL-2/IFN-γ ratio between ATB and LTBI was more obvious after 72h stimulation than after 24h, and achieved the highest AUC to discriminate ATB from LTBI, based on ROC analysis. These results may be due to a more adequate stimulation of central memory T-cell responses using a long-term stimulation assay[15]. Another interesting find was that PPD-induced IL-2/IFN-γ ratio showed higher diagnostic value than the ESAT-6/CFP-10-induced ratio. As a mixed antigen, PPD may provoke more comprehensive and diverse immune responses than ESAT-6/CFP-10[27]. Thus, PPD-stimulated assays may result in a more obvious difference and facilitate a better distinction between ATB and LTBI.

## 5. Conclusions

In conclusion, this study indicates that EC-stimulated release of IL-2 and IP-10 after short-term and long-term incubation represents a good diagnostic tool for distinction between TB-infected and TB-uninfected individuals. In addition, PPD-stimulated IL-2/IFN-γ ratio after 72h incubation had the diagnostic potential to discriminate between ATB and LTBI. These new biomarkers have been validated with high levels of accuracy in a prospective clinical-based cohort. We suggest that incorporation of these new biomarkers in immunodiagnostic assays may benefit the diagnosis of ATB and LTBI in future.

## References

[1] Barry CE 3rd, Boshoff HI, Dartois V, Dick T, Ehrt S, Flynn J, et al (2009) The spectrum of latent tuberculosis: rethinking the biology and intervention strategies. Nat Rev Microbiol 7: 845–855. 10.1038/nrmicro2236 19855401 PMC4144869

[2] Corbett EL, Watt CJ, Walker N, Maher D, Williams BG, Raviglione MC, et al (2003) The growing burden of tuberculosis: global trends and interactions with the HIV epidemic. Arch Intern Med 163: 1009–1021. 10.1001/archinte.163.9.1009 12742798

[3] Hur YG, Crampin AC, Chisambo C, Kanyika J, Houben R, Ndhlovu R, et al (2014) Identification of immunological biomarkers which may differentiate latent tuberculosis from exposure to environmental nontuberculous mycobacteria in children. Clin Vaccine Immunol 21: 133–142. 10.1128/CVI.00620-13 24285818 PMC3910933

[4] Walzl G, Ronacher K, Hanekom W, Scriba TJ, Zumla A (2011) Immunological biomarkers of tuberculosis. Nat Rev Immunol 11: 343–354. 10.1038/nri2960 21475309

[5] Pai M, Denkinger CM, Kik SV, Rangaka MX, Zwerling A, Oxlade O, et al (2014) Gamma interferon release assays for detection of Mycobacterium tuberculosis infection. Clin Microbiol Rev 27: 3–20. 10.1128/CMR.00034-13 24396134 PMC3910908

[6] Thillai M, Pollock K, Pareek M, Lalvani A (2014) Interferon-gamma release assays for tuberculosis: current and future applications. Expert Rev Respir Med 8: 67–78. 10.1586/17476348.2014.852471 24308653

[7] Moran Mendoza O (2011) Interferon-gamma release assays for the diagnosis of latent Mycobacterium tuberculosis infection. Eur Respir J 38: 1237–1238; author's reply 1238–1239. 10.1183/09031936.00182710 22045795

[8] Rangaka MX, Wilkinson KA, Glynn JR, Ling D, Menzies D, Mwansa-Kambafwile J, et al (2012) Predictive value of interferon-gamma release assays for incident active tuberculosis: a systematic review and meta-analysis. Lancet Infect Dis 12: 45–55. 10.1016/S1473-3099(11)70210-9 21846592 PMC3568693

[9] Metcalfe JZ, Everett CK, Steingart KR, Cattamanchi A, Huang L, Hopewell PC, et al (2011) Interferon-gamma release assays for active pulmonary tuberculosis diagnosis in adults in low- and middle-income countries: systematic review and meta-analysis. J Infect Dis 204 Suppl 4: S1120–1129. 21996694 10.1093/infdis/jir410 PMC3192542

[10] Mamishi S, Pourakbari B, Teymuri M, Rubbo PA, Tuaillon E, Keshtkar AA, et al (2014) Diagnostic accuracy of IL-2 for the diagnosis of latent tuberculosis: a systematic review and meta-analysis. Eur J Clin Microbiol Infect Dis 33: 2111–2119. 10.1007/s10096-014-2190-z 24993150

[11] Wang S, Diao N, Lu C, Wu J, Gao Y, Chen J, et al (2012) Evaluation of the diagnostic potential of IP-10 and IL-2 as biomarkers for the diagnosis of active and latent tuberculosis in a BCG-vaccinated population. PLoS One 7: e51338 10.1371/journal.pone.0051338 23272100 PMC3522729

[12] Ruhwald M, Aabye MG, Ravn P (2012) IP-10 release assays in the diagnosis of tuberculosis infection: current status and future directions. Expert Rev Mol Diagn 12: 175–187. 10.1586/erm.11.97 22369377

[13] Biselli R, Mariotti S, Sargentini V, Sauzullo I, Lastilla M, Mengoni F, et al (2010) Detection of interleukin-2 in addition to interferon-gamma discriminates active tuberculosis patients, latently infected individuals, and controls. Clin Microbiol Infect 16: 1282–1284. 10.1111/j.1469-0691.2009.03104.x 19886902

[14] Youden WJ (1950) Index for rating diagnostic tests. Cancer 3: 32–35. 15405679 10.1002/1097-0142(1950)3:1<32::aid-cncr2820030106>3.0.co;2-3

[15] Sallusto F, Lenig D, Forster R, Lipp M, Lanzavecchia A (1999) Two subsets of memory T lymphocytes with distinct homing potentials and effector functions. Nature 401: 708–712. 10.1038/44385 10537110

[16] Kabeer BS, Raja A, Raman B, Thangaraj S, Leportier M, Ippolito G, et al (2011) IP-10 response to RD1 antigens might be a useful biomarker for monitoring tuberculosis therapy. BMC Infect Dis 11: 135 10.1186/1471-2334-11-135 21595874 PMC3120672

[17] Ruhwald M, Dominguez J, Latorre I, Losi M, Richeldi L, Pasticci MB, et al (2011) A multicentre evaluation of the accuracy and performance of IP-10 for the diagnosis of infection with M. tuberculosis. Tuberculosis (Edinb) 91: 260–267. 21459676 10.1016/j.tube.2011.01.001

[18] Ruhwald M, Bodmer T, Maier C, Jepsen M, Haaland MB, Eugen-Olsen J, et al (2008) Evaluating the potential of IP-10 and MCP-2 as biomarkers for the diagnosis of tuberculosis. Eur Respir J 32: 1607–1615. 10.1183/09031936.00055508 18684849

[19] Azzurri A, Sow OY, Amedei A, Bah B, Diallo S, Peri G, et al (2005) IFN-gamma-inducible protein 10 and pentraxin 3 plasma levels are tools for monitoring inflammation and disease activity in Mycobacterium tuberculosis infection. Microbes Infect 7: 1–8. 10.1016/j.micinf.2004.09.004 15716076

[20] Whittaker E, Gordon A, Kampmann B (2008) Is IP-10 a better biomarker for active and latent tuberculosis in children than IFNgamma? PLoS One 3: e3901 10.1371/journal.pone.0003901 19065267 PMC2588495

[21] Goyal N, Kashyap B, Kaur IR (2016) Significance of IFN-/IL-2 Ratio as a Circulating Diagnostic Biomarker in Extrapulmonary Tuberculosis. Scand J Immunol 83: 338–344. 10.1111/sji.12424 26946082

[22] Nemeth J, Winkler HM, Zwick RH, Muller C, Rumetshofer R, Boeck L, et al (2012) Peripheral T cell cytokine responses for diagnosis of active tuberculosis. PLoS One 7: e35290 10.1371/journal.pone.0035290 22523581 PMC3327656

[23] Sester U, Fousse M, Dirks J, Mack U, Prasse A, Singh M, et al (2011) Whole-blood flow-cytometric analysis of antigen-specific CD4 T-cell cytokine profiles distinguishes active tuberculosis from non-active states. PLoS One 6: e17813 10.1371/journal.pone.0017813 21423578 PMC3058054

[24] Lanzavecchia A, Sallusto F (2000) Dynamics of T lymphocyte responses: intermediates, effectors, and memory cells. Science 290: 92–97. 11021806 10.1126/science.290.5489.92

[25] Caccamo N, Guggino G, Joosten SA, Gelsomino G, Di Carlo P, Titone L, et al (2010) Multifunctional CD4(+) T cells correlate with active Mycobacterium tuberculosis infection. Eur J Immunol 40: 2211–2220. 10.1002/eji.201040455 20540114

[26] Millington KA, Innes JA, Hackforth S, Hinks TS, Deeks JJ, Dosanjh DP, et al (2007) Dynamic relationship between IFN-gamma and IL-2 profile of Mycobacterium tuberculosis-specific T cells and antigen load. J Immunol 178: 5217–5226. 17404305 10.4049/jimmunol.178.8.5217 PMC2743164

[27] Sutherland JS, de Jong BC, Jeffries DJ, Adetifa IM, Ota MO (2010) Production of TNF-alpha, IL-12(p40) and IL-17 can discriminate between active TB disease and latent infection in a West African cohort. PLoS One 5: e12365 10.1371/journal.pone.0012365 20811496 PMC2927558

